# Effects of stroboscopic visual training on sport performance: a systematic review and meta-analysis with exploratory exposure–response modelling

**DOI:** 10.1186/s13102-026-01765-y

**Published:** 2026-07-21

**Authors:** Jianhao Lu, Meng Liu, Suyan Xu, Liang Zhang, Dapeng Bao, Zhipeng Xue, Lingzhi He, Yan Huang, Ke Bao, Olivier Girard

**Affiliations:** 1 Sports Coaching College, Beijing Sport University, Beijing, 100084 China; 2 University of Jinan, Jinan, 250022 China; 3 China Winter Sports College, Beijing Sport University, Beijing, 100084 China; 4 School of Strength and Conditioning, Beijing Sport University, Beijing, 100084 China; 5 China Institute of Sport and Health Science, Beijing Sport University, Beijing, 100084 China; 6 Medical Examination Center, Peking University Third Hospital, Beijing, 100191 China; 7 Key Laboratory for Performance Training and Recovery of General Administration of Sport, Beijing Sport University, Beijing, 100084 China; 8 Beijing Shichahai Sports School, No. 57 Di’anmen West Street, Xicheng District, Beijing, 100009 China; 9 School of Human Sciences, The University of Western Australia, Perth, Australia

**Keywords:** Intermittent vision, Perceptual-cognitive performance, Reaction time, Decision making, Sport-specific skills, Meta-analysis, Athletic performance

## Abstract

**Background:**

Stroboscopic visual training (SVT) intermittently occludes vision during skill execution to increase perceptual‑cognitive demand and improve information pickup. Evidence in sport is heterogeneous, and key exposure parameters are inconsistently reported; therefore, we quantified SVT effects and explored whether a duty‑cycle‑adjusted exposure metric relates to performance change.

**Methods:**

PubMed, Web of Science, Scopus, SPORTDiscus, Embase and Cochrane CENTRAL were searched from inception to 19 August 2025 for controlled studies comparing SVT with non‑stroboscopic controls in athletes or physically active participants. Outcomes were grouped as perceptual‑cognitive, visuomotor reaction time, motor speed/reactive agility, or visual tracking. Random-effects meta-analysis (REML) computed pooled Hedges’ g. Because some studies contributed more than one comparison-level effect size, a multilevel random-effects model with comparisons nested within studies was fitted as a robustness check, and variance partitioning was used to describe the relative contribution of within-study versus between-study heterogeneity. Analyses were stratified by exposure timescale (acute single‑session vs multi‑session programmes). Exposure–response modelling used occlusion‑minutes (training minutes × opaque duty‑cycle fraction) when dose data were available.

**Results:**

Seventeen studies (513 participants; 22 comparison-level effect sizes) were included. Overall, SVT improved sport-related outcomes (g = 0.79, 95% CI 0.39–1.19; I^2^ = 84.3%; 95% prediction interval − 1.02 to 2.60). A multilevel random-effects model yielded a similar pooled estimate (g = 0.84, 95% CI 0.33–1.34; *p* = 0.001) and suggested that heterogeneity was predominantly between studies. Benefits were evident for multi-session programmes (15 studies; k = 20; g = 0.88, 95% CI 0.45–1.32) but not for acute exposures (2 studies; k = 2; g = 0.06, 95% CI − 0.26 to 0.38). Pooled effects were largest for perceptual-cognitive tasks (3 studies; k = 3; g = 1.23) and visuomotor reaction time (11 studies; k = 12; g = 0.99), and smaller for motor speed / reactive agility (6 studies; k = 6; g = 0.35). In the multi-session reaction-time subset with dose data (5 studies; k = 5; occlusion-minutes 94.9–325.2), occlusion-minutes were not associated with effect size (linear meta-regression slope = − 0.0016 g per occlusion-minute, 95% CI − 0.0058 to 0.0025; *p* = 0.503). Publication bias analyses did not suggest small-study effects (Egger *p* = 0.289; trim-and-fill: 0 studies imputed).

**Conclusions:**

SVT shows a moderate-to-large average benefit across heterogeneous designs, driven primarily by multi-session programmes. Evidence is stronger for near-to-intermediate transfer outcomes than for acute effects or far transfer to representative sport performance. Current data are insufficient to define an optimal SVT dose based on occlusion-minutes alone. Future trials should standardise device reporting, use conceptually grounded transfer outcomes, and include delayed follow-up assessments.

**Trial registration:**

PROSPERO CRD420251090541.

**Supplementary Information:**

The online version contains supplementary material available at https://doi.org/10.1186/s13102-026-01765-y.

## Key points

SVT improves sport-related outcomes; however, because the pooled estimate combines acute and multi-session exposures, it reflects an overall stroboscopic exposure effect rather than a pure training-adaptation effect. When stratified by timescale, multi-session programmes showed clear benefits (g ≈ 0.88), whereas acute single-session exposures showed no clear effect (g ≈ 0.06).

Effects were largest for perceptual–cognitive outcomes and visuomotor reaction time. In contrast, effects for motor speed / reactive agility were smaller, although still positive on average. Evidence for visual tracking and multiple-object tracking (MOT) remains limited because it is currently based on a single study.

In multi-session reaction-time outcomes with extractable dose data (5 studies; k = 5), exposure-response modelling showed no association between occlusion-minutes and effect size (linear slope = −0.0016 g per occlusion-minute; p = 0.503). Given the small dose-informative subset, current evidence does not support inference about thresholds or an optimal dose.

**Supplementary Information:**

The online version contains supplementary material available at https://doi.org/10.1186/s13102-026-01765-y.

## Introduction

Vision is a primary sensory channel supporting athletic performance, particularly in dynamic sports where athletes must detect, prioritise, and act on rapidly changing information. Consistent with this role, expert athletes typically outperform less-skilled counterparts in several perceptual-cognitive and visuomotor domains, including more efficient gaze behaviour, faster decision processes, and more effective use of advance cues [1–4]. These observations help explain why training approaches targeting perceptual and decision processes continue to attract sustained interest in sport science.

Traditional vision training has often focused on static visual functions (e.g., acuity, contrast sensitivity, accommodation) [5]. While these functions are important prerequisites, they are rarely sufficient for performance in fast, information-rich sport environments, where athletes must interpret complex scenes, anticipate trajectories, and select actions under time pressure [6]. Related work also suggests that elite performers can rapidly learn complex dynamic visual scenes, which reinforces the importance of training approaches that challenge vision under realistic constraints [7].

Stroboscopic visual training (SVT) is one such intervention. SVT uses eyewear that alternates between transparent and opaque states to intermittently restrict visual information during task execution. This intermittent occlusion is intended to increase reliance on predictive processing, more selective sampling of task-relevant cues, and more efficient visuomotor control under informational constraints [6, 8]. Proposed mechanisms include improved selective attention, reduced dependence on continuous visual feedback, and stronger feedforward control through better anticipation of partially occluded events [9–14]. However, mechanistic inferences in applied sport settings remain limited because most trials rely on behavioural outcomes rather than direct neurophysiological measures [15].

Despite enthusiasm for SVT, the empirical literature is methodologically diverse. Studies vary widely in sport context, participant level, programme duration, session frequency, stroboscopic timing parameters, and the extent to which training drills match the informational constraints of the target performance task [6, 16]. Outcomes also differ substantially, spanning proximal perceptual-cognitive tests, intermediate visuomotor reaction-time measures, and more distal sport-specific skills. This diversity complicates synthesis and increases the risk of over-generalising across outcomes that may reflect meaningfully different transfer distances.

This challenge is amplified by ongoing debate about whether sports vision training produces meaningful far transfer to sport performance. Recent syntheses have highlighted heterogeneity in study designs and limitations in outcome representativeness across the broader sports-vision literature [17, 18]. In parallel, perspective and correspondence articles in *Sports Medicine* have argued both for caution (i.e., limited evidence for far transfer from generalised training) and for a more nuanced interpretation of an evolving evidence base [19–21]. Frameworks such as the Modified Perceptual Training Framework emphasise that training and testing tasks should be classified by representativeness and information–movement coupling, rather than treated as interchangeable indicators of sport performance [22]. Together, these contributions imply that meta-analyses should be explicit about which outcomes are synthesised, what kind of transfer is being inferred, and which design features limit comparability across trials.

Existing systematic reviews and meta-analyses on SVT and sports vision training have generally reported positive effects, but also substantial heterogeneity and inconsistent reporting of device parameters and exposure metrics [6, 15, 16, 23, 24]. Formal exploration of exposure–response relationships has been uncommon, and commonly reported “dose” variables may fail to capture critical features such as timing structure, task constraints, and decision complexity. Therefore, a synthesis that is transparent about design heterogeneity, cautious about exposure inference, and explicit about estimands and transfer claims remains needed.

Accordingly, this systematic review and meta-analysis aimed to: (1) quantify the overall effect of SVT on sport-related outcomes; (2) estimate domain-specific effects for perceptual-cognitive tasks, visuomotor reaction time, motor speed/reactive agility, and visual tracking; (3) explore exposure–response relationships using a duty-cycle-adjusted occlusion exposure metric while acknowledging its conceptual limitations; and (4) assess evidence certainty and risk of bias using established tools.

## Methods

### Protocol and registration

This review was designed a priori, reported according to the PRISMA 2020 checklist [25], and prospectively registered on PROSPERO (CRD420251090541; registered 5 January 2025). The full protocol is provided in Supplementary Appendix A, including post-hoc deviations: (i) the addition of exploratory exposure–response modelling and (ii) the inclusion of single-session (acute) trials as mechanistic evidence in overall pooling, while excluding them from exposure–response modelling and from retention-focused interpretations.

### Searches

A medical librarian assisted in developing the search strategy. Six databases – PubMed, Web of Science, Scopus, SPORTDiscus (EBSCOhost), Embase, and Cochrane CENTRAL – were searched from inception to 18 July 2025, with an update on 19 August 2025. A surveillance search on 1 February 2026 identified no additional eligible controlled studies and did not alter the evidence base or PRISMA study counts. Trial registries (ClinicalTrials.gov and the Chinese Clinical Trial Registry [ChiCTR]) were searched in parallel but were not included in PRISMA database counts.

The core concept string targeted SVT and sport-performance outcomes: (stroboscopic OR stroboscope* OR strobe* OR intermittent vision OR shutter glasses OR stroboscopic eyewear) AND (vision* OR visu* OR percept* OR motor* OR reaction* OR decision* OR performance OR sport*). Device-specific terms (e.g., Senaptec, Nike Vapor, PLATO) and field tags were appended where supported (full strategies in Supplementary File 1). No restrictions were placed on language, publication date, or document type. Additional sources included the first 200 Google Scholar titles, backward citation searching, and Web of Science forward citation tracking.

### Eligibility criteria (PICOS)

Eligibility criteria are summarised in Table 1:

**Table 1:** Eligibility criteria based on the PICOS framework

Component	Inclusion criteria	Exclusion criteria
Participants	Healthy adolescents/adults (≥ 10 y) who were athletes or physically active participants in sport related contexts (including training or sport relevant tasks)	Clinical or rehabilitative populations; para athletes
Interventions	Wearable stroboscopic/shutter eyewear providing intermittent visual occlusion during task execution, delivered as either (a) multi session programmes (≥ 1 week) or (b) single session acute exposures. Device settings (e.g., duty cycle, on/off timing, frequency, progression) were extracted when reported. Acute trials were eligible for overall pooling but were excluded from exposure–response modelling and from retention-focused interpretations	Non stroboscopic visual drills; VR/AR interventions; multimodal sensory interventions where the stroboscopic component could not be isolated
Comparators	Non stroboscopic control conditions, including passive wait list, sham/transparent eyewear, or conventional training without stroboscopic occlusion	No control group, or an active comparator that was stroboscopic and therefore not a non stroboscopic control for the primary contrast
Outcomes	Perceptual cognitive, visuomotor, and sport related performance outcomes (e.g., reaction time, accuracy, agility/reactive agility, sport skill performance), assessed immediately post intervention (acute studies) or within ≤ 7 days after intervention completion (multi session studies), with sufficient data to compute Hedges' g	Outcomes not plausibly related to sport performance; insufficient extractable data (e.g., % change only without variability)
Study design	RCTs, quasi RCTs, and controlled trials. In cross over designs, only first period data were used	Uncontrolled studies; qualitative studies; reviews; conference abstracts without sufficient data

*PICOS* Participants, Interventions, Comparators, Outcomes, Study design

### Study selection

**Fig. 1:**
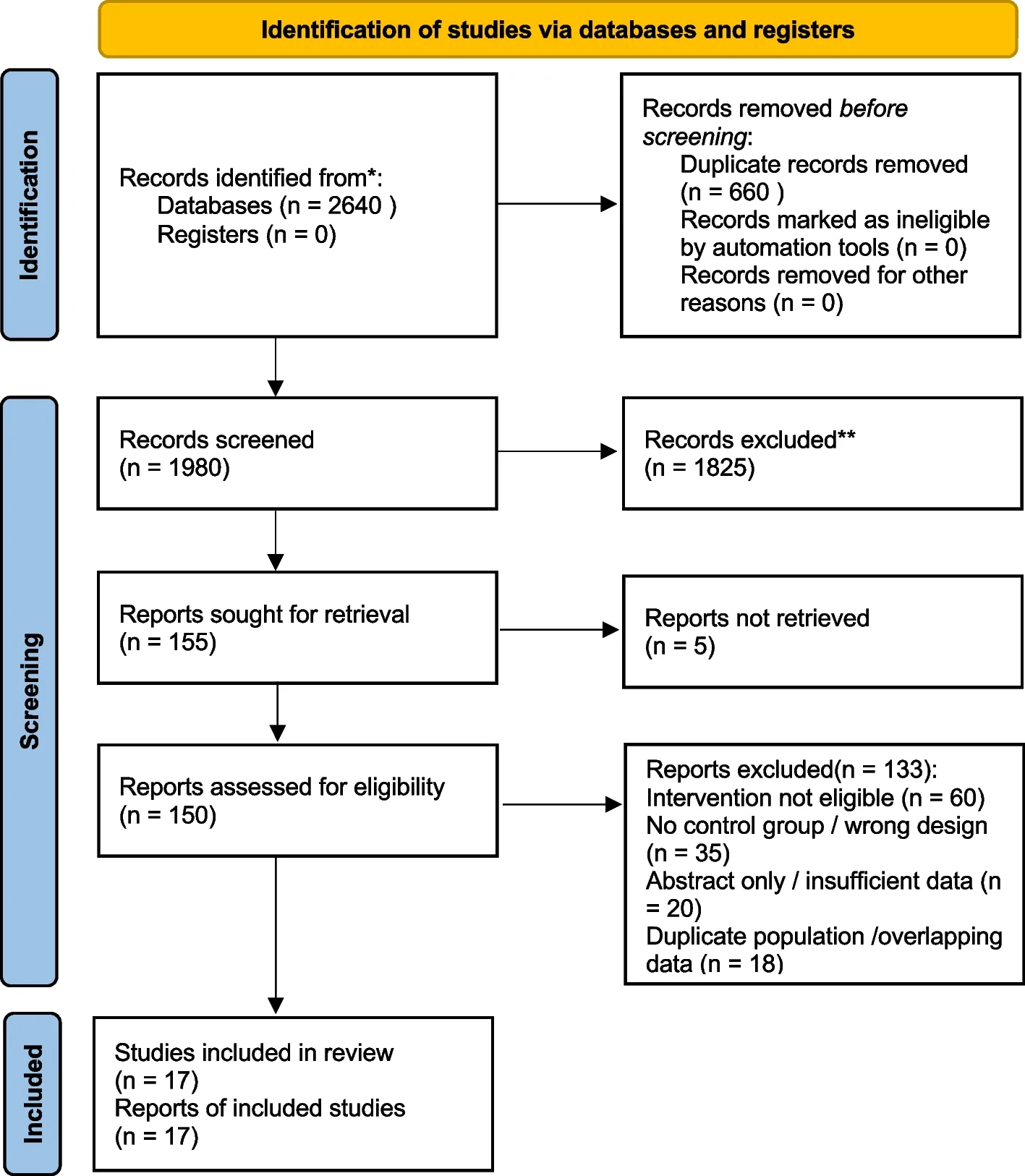
PRISMA 2020 flow diagram of study selection. (Records identified, screened, assessed, and included as described above.)

All records were imported into Rayyan for duplicate detection (automatic and manual) [26]. Two reviewers (LM and LJH) independently screened titles/abstracts and assessed full texts against the prespecified PICOS criteria. A calibration exercise on 20% of randomly selected citations yielded substantial inter-reviewer agreement (κ = 0.86) [27]. Disagreements were resolved by discussion or, if necessary, by consulting a third reviewer (XSY). Reasons for full-text exclusion are detailed in Supplementary File 2. The process is illustrated in the PRISMA flow diagram (Fig. 1).

### Data extraction and coding

A structured extraction sheet was piloted on two studies and then applied by the same pair of reviewers to extract five categories of information: (1) bibliographic details (author, year, country, sport); (2) participant characteristics; (3) intervention characteristics (programme duration, sessions·week⁻^1^, session length, and device settings); (4) outcome data (post‑test mean, standard deviation, and sample size); and (5) study design features.

When studies reported multiple eligible outcomes, all were extracted and then classified by outcome domain. When studies included multiple SVT arms that shared a single control group, we avoided double‑counting the control group by splitting the control group sample size evenly across comparisons, consistent with standard guidance [28]. For cross‑over trials, only first‑period data were extracted to minimise carryover bias [28]. When studies reported multiple time points, we extracted the post‑test outcome closest to the intervention endpoint (within ≤ 1 week). Two datasets were digitised using WebPlotDigitizer (v4.5) [29]. For missing data, authors were contacted twice (≤ 7 days apart); non‑responses are documented in Table S2.

Effect‑direction harmonisation. Effect sizes were coded so that positive values consistently indicated better performance with SVT. For outcomes where lower scores indicate better performance (e.g., reaction time or execution time), the effect direction was sign‑reversed so that faster times corresponded to positive Hedges’ g.

An exposure‑timescale variable was coded to define the estimand and guide exposure–response modelling (acute = single session; multi‑session = ≥ 1 week). For exposure–response analyses based on occlusion‑minutes, comparisons lacking duty‑cycle information were retained in the overall meta‑analysis but excluded from occlusion‑minutes modelling because exposure could not be quantified on that scale. Throughout, “study” or “trial” refers to a unique included study, whereas k denotes the number of comparison-level effect sizes contributing to a given analysis.

### Risk-of-bias assessment

Randomised trials were evaluated with RoB 2 [30] and non-randomised trials with ROBINS-I [31]. Two assessors (LM and LJH) piloted both tools on three studies, refined decision rules, and then independently rated the remaining trials. Disagreements were resolved by consensus; one case required arbitration by a third reviewer (XSY). Overall judgements followed the “worst-domain” principle for each tool. Risk-of-bias plots were produced with robvis (Figures S4–S5) [32].

### Statistical analysis

All analyses were conducted in R 4.4 using metafor and orchaRd [33–35]. Effect sizes were expressed as Hedges’ g (positive = SVT superior) [36]. When studies reported SE or IQR, means and SDs were imputed using Wan et al. [37].

#### Pooled effects, heterogeneity, and estimand clarification

Overall effects were estimated using a conventional random-effects model (REML), with heterogeneity summarised using τ^2^ and I^2^ [38]. We also report 95% prediction intervals for the conventional random-effects model [39]. Because some studies contributed multiple comparison-level effect sizes, a multilevel random-effects model (comparisons nested within studies) was fitted as a robustness check. For this multilevel model, we report the level-2 (within-study) and level-3 (between-study) variance components, along with the ICC (σ^2^_L3/(σ^2^_L2 + σ^2^_L3)) to describe the proportion of modelled heterogeneity attributable to between-study differences. As a further sensitivity analysis, CR2 cluster-robust inference was examined [40–42].

The primary overall pooling included both acute and multi-session exposures; therefore, pooled estimates were interpreted as overall stroboscopic exposure effects rather than retention-only training effects. To clarify the estimand, we also synthesised acute versus multi-session comparisons separately.

#### Exposure metric and non-linear exposure–response modelling

To operationalise stroboscopic occlusion exposure, a composite metric was computed as:

$$\begin{aligned}\mathrm{occlusion}&-\mathrm{minutes}\;=\;\mathrm{total}\;\mathrm{training}\;\mathrm{minutes}\\&\;\times\;\mathrm{opaque}\;\mathrm{duty}-\mathrm{cycle}\;\mathrm{fraction}\end{aligned}$$

where *total training minutes* represents the cumulative duration of SVT exposure across the intervention period, and the *opaque duty-cycle fraction* represents the proportion of each stroboscopic cycle during which the lenses were opaque.

When on/off timing was reported, the opaque duty-cycle fraction was calculated as:

$$\mathrm{opaque}\;\mathrm{duty}-\mathrm{cycle}\;\mathrm{fraction}\;=\;\mathrm{off}\;/\;(\mathrm{on}\;+\;\mathrm{off})$$

where *off* denotes the opaque interval and *on* the transparent interval. If directly reported, the opaque duty-cycle fraction was used as provided.

This composite metric captures cumulative time spent under opaque lenses but does not represent the temporal structure of the stroboscopic sequence beyond the overall duty cycle (e.g., on/off frequency or changes in frequency over time). It also does not reflect task constraints that can materially alter training demand, such as decision complexity, stimulus speed, task representativeness, or information-movement coupling. Accordingly, occlusion-minutes was treated as an exploratory proxy for exposure rather than as a definitive measure of SVT “dose”.

Exposure–response analyses were prespecified for multi-session programmes and, within these, for reaction-time comparisons with available occlusion-minutes. Non-linear associations were planned using restricted cubic spline meta-regression (three knots at the 10th, 50th, and 90th percentiles) [43]. Restricted cubic spline models were fitted only when the number of dose-informative comparisons was sufficient (prespecified k ≥ 8) and knot locations were distinct; otherwise, linear meta-regression was used to avoid unstable non-linear estimates.

#### Influence diagnostics, sensitivity analyses, and publication bias

Influence was evaluated via standard diagnostics and leave-one-out procedures [44]. For meta-analyses with k ≥ 10, funnel plots and Egger’s regression (α = 0.10) assessed asymmetry [45], with trim-and-fill applied exploratorily [46].

### Certainty of evidence

Two reviewers independently rated outcome-level certainty using the GRADE framework [47]. Summary ratings are reported in Supplementary Table S3.

### Use of large language models (LLMs)

During manuscript preparation, a large language model (ChatGPT, OpenAI) was used solely for language polishing and checklist alignment. No AI tools were used to generate, analyse, or interpret data or images, nor to create or modify figures. All authors reviewed and approved the manuscript and take full responsibility for its integrity and accuracy.

## Results

### Study selection

Database searches to 19 August 2025 identified 2,640 records: PubMed (340), Web of Science (988), Scopus (673), SPORTDiscus (16), Embase (470), and Cochrane CENTRAL (153) (see Supplementary File 1). After removing 660 duplicates in Zotero, 1,980 records were dual-screened in Rayyan, excluding 1,825. Full texts were retrieved for 155 records (five unobtainable), leaving 150 articles assessed for eligibility. Seventeen studies met all criteria and were included in the meta-analysis (Fig. 1). A surveillance search on 1 February 2026 identified no additional eligible controlled studies. Reasons for the 133 full-text exclusions are detailed in Supplementary File 2.

### Study characteristics

Seventeen trials from ten countries enrolled 513 participants engaged in sport-related training (Table 2). Sample sizes ranged from 6 to 62 per trial (median = 30). Study designs included 11 parallel-group RCTs, 2 quasi-RCTs, 1 randomised cross-over trial (acute exposure), and 3 non-randomised controlled comparisons. Among the 14 randomised trials, 6 were rated low risk and 8 raised some concerns (RoB 2), with none rated high risk. The three non-randomised studies were judged moderate risk across ROBINS-I domains.

**Table 2:** Key study characteristics (*n* = 17 trials)

Study ID	Country	Population/sport context	N (IG/CG)	Design	Overall risk of bias	Weeks	Total training time (min)
WilkinsNelson_2018 [48]	UK	Youth football goalkeepers	3/3	NRCT	Moderate (ROBINS I)	7	635
WilkinsGray_2015 [9]	UK	Catching/interceptive task	15/15	RCT	Low (RoB 2)	5	165
Zwierko_2024 [49]	Poland	Women volleyball	13/12	RCT	Low (RoB 2)	6	495
Fortes_2023 [50]	Brazil	Professional men soccer	14/14	RCT	Low (RoB 2)	8	480
Zwierko_2023 [51]	Poland	Youth volleyball	25/25	RCT	Low (RoB 2)	6	270
ZwierkoBlock_2024 [52]	Poland	Youth women volleyball (blocking)	13/12	RCT	Low (RoB 2)	6	495
Gao_2024 [53]	China	Elite shooters	13/13	RCT	Low (RoB 2)	8	392
Appelbaum_2011 [54]	USA	Varsity football + laboratory tasks	20/21	RCT	Some concerns (RoB 2)	2	225
Boiangin_2019 [55]	USA	Tennis (anticipation)	20/10	RCT	Some concerns (RoB 2)	1	180
ZwierkoSoccerRA_2024 [56]	Spain	Soccer reactive agility (acute)	22/22	Randomised cross over	Some concerns (RoB 2)	0	8
Hulsdunker_2021 [57]	Germany	Elite youth sport (visuomotor)	16/16	Quasi RCT	Some concerns (RoB 2)	10	450
VasileClimb_2024 [58]	Romania	Youth climbing	9/8	NRCT	Moderate (ROBINS I)	52	NR
SmithMitroff_2012 [59]	USA	Laboratory timing task	15/15	RCT	Some concerns (RoB 2)	2	7
Ellison_2020 [60]	UK	Active males (acute)	31/31	RCT	Some concerns (RoB 2)	0	7
Palmer_2022 [61]	Australia	Youth soccer (dribbling)	18/18	RCT	Some concerns (RoB 2)	4	80
Oudejans_2012 [62]	Netherlands	Elite women basketball	6/15	NRCT	Moderate (ROBINS I)	12	224
Hulsdunker_2019 [63]	Germany	Top level badminton	5/5	Quasi RCT	Some concerns (RoB 2)	4	480

*Abbreviations IG* Intervention group, *CG* Control group, *NR* Not reported, *NRCT* Non-randomised controlled trial

Two trials delivered single-session (acute) exposures, while the remainder implemented multi-session programmes lasting 1–52 weeks (most lasted 1–10 weeks). Across studies reporting total training minutes (16/17), total training time ranged from 7 to 635 min (median ≈ 248 min). The most commonly used devices were Senaptec Strobe (8 trials) and Nike Vapor strobe eyewear (6 trials), with PLATO goggles (2 trials) and MJ Impulse (1 trial) less frequently used (Table 3).

**Table 3:** Stroboscopic intervention parameters and comparators (as reported; derived schedule metrics indicated)

Study ID	Device	Strobe parameters (as reported)	Weeks	Sessions/week	Total minutes	Avg minutes/session*	Comparator used for meta analysis
WilkinsNelson_2018 [48]	Senaptec Strobe	Frequency progression (reported); duty cycle reported (~ 50%)	7	2.0	635	45.4	Normal training/normal vision (non stroboscopic)
WilkinsGray_2015 [9]	PLATO	On/off timing reported (range)	5	2.0	165	16.5	Normal vision control (non stroboscopic)
Zwierko_2024 [49]	Senaptec Strobe	15- > 9 Hz; duty cycle ~ 50%	6	3.0	495	27.5	Regular training/normal vision (non stroboscopic)
Fortes_2023 [50]	Nike Vapor	On/off timing reported	8	3.0	480	20.0	Regular training/normal vision (non stroboscopic)
Zwierko_2023 [51]	Senaptec Strobe	15- > 9 Hz; duty cycle ~ 50%	6	5.0	270	9.0	Regular training/normal vision (non stroboscopic)
ZwierkoBlock_2024 [52]	Senaptec Strobe	15- > 9 Hz; duty cycle ~ 50%	6	3.0	495	27.5	Regular training/normal vision (non stroboscopic)
Gao_2024 [53]	Senaptec Strobe	Frequency progression reported; duty cycle ~ 50%	8	2.0	392	24.5	Regular training/normal vision (non stroboscopic)
Appelbaum_2011 [54]	Nike Vapor	Frequency/level progression reported	2	5.0	225	22.5	Transparent/sham eyewear (non stroboscopic)
Boiangin_2019 [55]	Nike Vapor	On/off timing reported	1	6.0	180	30.0	Placebo/sham eyewear (non stroboscopic)
ZwierkoSoccerRA_2024 [56]	Senaptec Strobe	15 Hz; duty cycle ~ 50% (acute)	0	1.0	8	8.0	Normal vision (non stroboscopic)
Hulsdunker_2021 [57]	Senaptec Strobe	15- > 8 Hz; duty cycle reported (50–70%)	10	2.0	450	22.5	Regular training/normal vision (non stroboscopic)
VasileClimb_2024 [58]	Senaptec Strobe	Frequency range reported; duty cycle reported (50–70%)	52	0.38	NR	NR	Regular training/normal vision (non stroboscopic)
SmithMitroff_2012 [59]	Nike Vapor	On/off timing reported (~ 4 Hz)	2	1.0	7	3.5	Transparent/sham eyewear (non stroboscopic)
Ellison_2020 [60]	Nike Vapor	On/off timing reported (~ 4 Hz) (acute)	0	1.0	7	7.0	Normal vision (non stroboscopic)
Palmer_2022 [61]	Nike Vapor	Device level progression reported	4	1.0	80	20.0	Normal vision (non stroboscopic)
Oudejans_2012 [62]	PLATO	Manual occlusion timing reported	12	1.5	224	12.4	Regular training/normal vision (non stroboscopic)
Hulsdunker_2019 [63]	MJ Impulse	Frequency progression reported; duty cycle reported (50–70%)	4	3.0	480	40.0	Regular training/normal vision (non stroboscopic)

^*^Avg minutes/session were derived as total minutes/(weeks × sessions/week) for multi-session programmes when all components were available; NR indicates not reportable from the published information

Outcomes were grouped into four domains: visuomotor reaction time (11 studies; k = 12 comparison-level effect sizes), motor speed/reactive agility (6 studies; k = 6), perceptual-cognitive tasks (3 studies; k = 3), and visual tracking/MOT (1 study; k = 1).

### Overall effect

Across 22 comparison-level effect sizes from 17 studies, a conventional random-effects model estimated using restricted maximum likelihood (REML) yielded a pooled Hedges’ g of 0.79 (95% CI 0.39–1.19; *p* < 0.001). Heterogeneity was substantial (τ^2^ = 0.71; I^2^ = 84.3%), and the 95% prediction interval was wide (− 1.02 to 2.60), indicating considerable variation in true effects across sports, outcome tasks, and implementation contexts. Because some studies contributed multiple comparisons, a multilevel random-effects model (comparisons nested within studies) was fitted. The multilevel pooled estimate was similar (g = 0.84; 95% CI 0.33–1.34; *p* = 0.001). The within-study variance component was estimated near the boundary (σ^2^_L2 ≈ 0.00), whereas the between-study variance component was substantial (σ^2^_L3 = 0.98), corresponding to an ICC of ~ 1.00. This indicates that heterogeneity was predominantly between studies rather than within studies. An additional CR2 sensitivity analysis yielded similar inference. Because the overall analysis combined acute and multi-session exposures, this estimate is interpreted as an overall stroboscopic exposure effect rather than as evidence of retained long-term training adaptation.

### Domain-specific effects

Domain-specific pooled effects were largest for perceptual-cognitive tasks (3 studies; k = 3; g = 1.23; 95% CI 0.73–1.73), followed by visuomotor reaction time (11 studies; k = 12; g = 0.99; 95% CI 0.22–1.76). Motor speed/reactive agility showed a smaller but statistically significant effect (6 studies; k = 6; g = 0.35; 95% CI 0.02–0.68). Only one study contributed to visual tracking/multiple-object tracking (MOT) (1 study; k = 1; g = 0.98; 95% CI 0.32–1.63), precluding estimation of between-study heterogeneity for this domain.

### Acute vs multi-session (estimand-clarifying analysis)

**Fig. 2:**
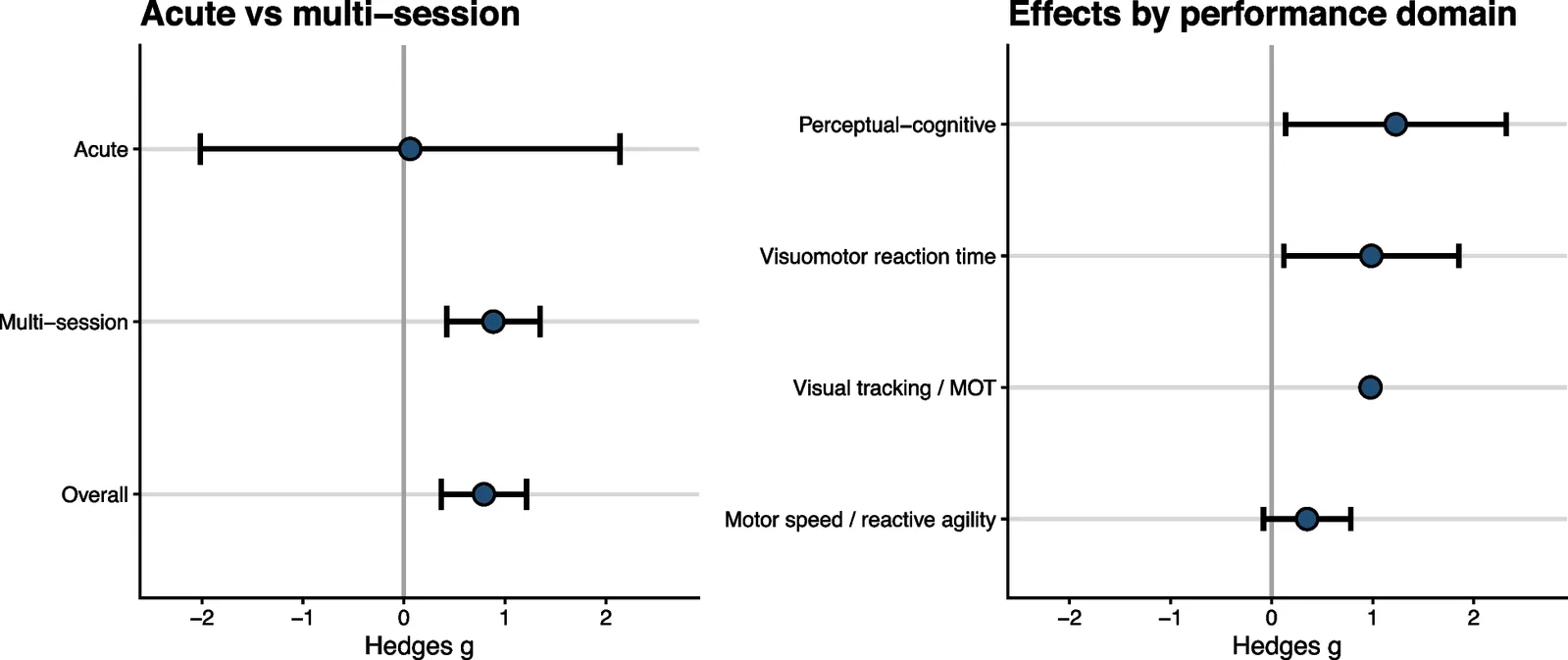
Summary meta-analytic effects of stroboscopic visual training (SVT) by exposure timescale and performance domain. A Pooled Hedges’ g for the overall synthesis, multi-session programmes, and acute single-session exposures. B Pooled effects by outcome domain: visual tracking/multiple-object tracking (MOT), perceptual-cognitive tasks, visuomotor reaction time, and motor speed/reactive agility. Points indicate pooled effect estimates and horizontal bars indicate 95% confidence intervals from random-effects models (REML). The vertical dashed line at g = 0 indicates no difference between SVT and control. Positive values indicate superior performance with SVT. k denotes the number of comparison-level effect sizes contributing to each estimate; where k = 1, the point represents the single available comparison

To clarify the estimand, we stratified comparisons by exposure timescale. Acute single-session exposures (2 studies; k = 2) showed no clear effect (g = 0.06; 95% CI − 0.26 to 0.38; *p* = 0.702). In contrast, multi-session programmes (15 studies; k = 20) showed a clear benefit (g = 0.88; 95% CI 0.45–1.32; p < 0.001). This pattern indicates that the overall pooled estimate is driven primarily by multi-session interventions, with acute findings reflecting mechanistic responses rather than retained training adaptation. Stratified and domain-specific pooled effects are shown in Fig. 2, with key pooled estimates summarised in Table 4.

**Table 4:** Summary meta-analytic results for stroboscopic visual training effects on sport performance

Analysis	k	Pooled g (95% CI)	p	Heterogeneity summary	95% PI
Panel A. Overall effect					
Overall (conventional random-effects; REML)	22	0.79 (0.39 to 1.19)	< 0.001	τ^2^ = 0.71; I^2^ = 84.3%	− 1.02 to 2.60
Overall (multilevel random-effects; comparisons nested within studies)	22	0.84 (0.33 to 1.34)	0.001	σ^2^L₂ ≈ 0.00; σ^2^L₃ = 0.98; ICC ≈ 1.00	NA
Overall (cluster-robust SE; CR2 robustness analysis)	22	0.79 (0.34 to 1.25)	0.002	NA	NA
Panel B. Effects by training time scale					
Acute single-session exposure effect	2	0.06 (− 0.26 to 0.38)	0.702	τ^2^ = 0.00; I^2^ = 0.0%	− 2.01 to 2.14
Multi-session training effect	20	0.88 (0.45 to 1.32)	< 0.001	τ^2^ = 0.77; I^2^ = 83.3%	− 1.01 to 2.78
Panel C. Effects by performance domain					
Perceptual-cognitive	3	1.23 (0.73 to 1.73)	< 0.001	τ^2^ = 0.00; I^2^ = 0.0%	− 1.99 to 4.45
Visuomotor reaction time	12	0.99 (0.22 to 1.76)	0.012	τ^2^ = 1.63; I^2^ = 90.5%	− 1.99 to 3.96
Motor speed/reactive agility	6	0.35 (0.02 to 0.68)	0.037	τ^2^ = 0.08; I^2^ = 47.2%	− 0.55 to 1.25
Visual tracking/MOT	1	0.98 (0.32 to 1.63)	0.003	NE	NE

k refers to the number of comparison-level effect sizes contributing to each analysis. Prediction intervals are reported for the conventional random-effects models where estimable, but are not reported for the multilevel robustness model or the CR2 robustness analysis. The multilevel model is reported with level-2 and level-3 variance components and the ICC. For the visual tracking/MOT domain, only one comparison contributed, so heterogeneity and prediction intervals were not estimable

*Abbreviations CI* Confidence interval, *CR2* Cluster-robust variance estimation with small-sample correction, *ICC* Intraclass correlation coefficient, *I*^*2*^ Inconsistency, *k* number of comparison-level effect sizes, *MOT* Multiple-object tracking, *NA* Not applicable, *NE* Not estimable, *PI* Prediction interval, *REML* Restricted maximum likelihood, *SE* Standard error, *SVT* Stroboscopic visual training; τ^2^, between-comparison variance

### Exposure–response analysis (occlusion-minutes)

**Fig. 3:**
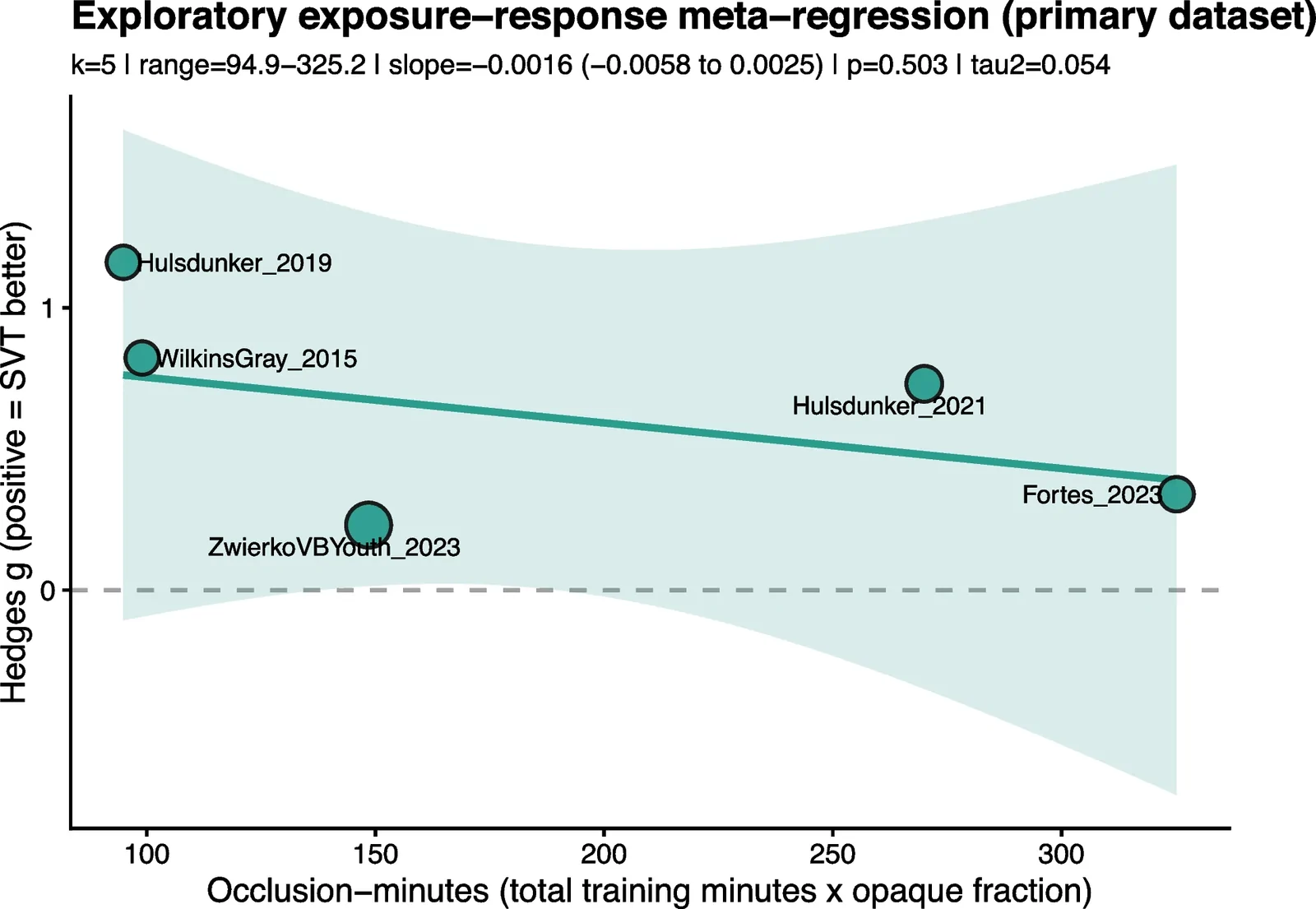
Exploratory exposure-response meta-regression of occlusion exposure and effect size in multi-session reaction-time outcomes. Scatter plot of comparison-level Hedges’ g values against occlusion-minutes (total training minutes × opaque duty-cycle fraction) for multi-session visuomotor reaction-time comparisons with extractable dose information (5 studies; k = 5; occlusion-minutes range 94.9–325.2). Bubble size is proportional to the random-effects inverse-variance weight. The solid line shows the fitted random-effects linear meta-regression (REML), with the shaded band indicating the 95% confidence interval. The dashed horizontal line denotes no effect (g = 0). Positive Hedges’ g indicates superior performance with SVT. Non-linear spline models were not fitted due to limited dose reporting (k = 5)

Occlusion exposure was quantified as occlusion-minutes (total training minutes × opaque duty-cycle fraction). Within multi-session visuomotor reaction-time comparisons, only a subset provided sufficient details to compute occlusion-minutes. These comparisons formed the primary dose-informative dataset (5 studies; k = 5; occlusion-minutes range 94.9–325.2). Random-effects meta-regression showed no association between occlusion-minutes and effect size (slope = − 0.0016 g per occlusion-minute; 95% CI − 0.0058 to 0.0025; *p* =0.503; τ^2^ = 0.0536). The exposure–response meta-regression is shown in Fig. 3, with full outputs for both the primary and sensitivity dose datasets reported in Table 5 (Panel B).

**Table 5:** Additional analyses (publication bias, exposure–response, and heterogeneity attenuation)

Item	Dataset	Result
Panel A. Publication-bias assessments (overall meta-analysis; k = 22)		
Egger’s regression (intercept test)	Overall	Intercept = − 0.50 (SE 0.46) t = − 1.09; df = 20; * p* = 0.289; 95% CI − 1.47 to 0.46
Trim-and-fill	Overall	k0 = 0 Adjusted g = 0.79 (0.39 to 1.19)
Panel B. Exploratory exposure–response modelling (outcome = visuomotor RT; multi-session; dose = occlusion-minutes)		
Exposure–response meta-regression (linear)	Primary dataset (k = 5)	Slope = − 0.0016 g per occlusion-minute (95% CI − 0.0058 to 0.0025) *p* = 0.503; τ^2^ = 0.054; dose range 94.9–325.2
Exposure–response meta-regression (linear; sensitivity)	Sensitivity dataset (k = 6)	Slope = − 0.0069 g per occlusion-minute (95% CI − 0.0140 to 0.0001) *p* = 0.126; τ^2^ = 0.717; dose range 4.2–325.2
Exposure–response meta-regression (restricted cubic spline)	Dose dataset (k = 5)	Not fitted (k < 8; prespecified threshold for spline modelling)
Panel C. Heterogeneity attenuation (visuomotor RT; multi-session)		
Random-effects model (before restriction)	Dose dataset (k = 6)	g = 1.09 (− 0.13 to 2.31); * p* = 0.069 τ^2^ = 1.184; I^2^ = 89.0%
Random-effects model (after restriction)	Primary subset (k = 5)	g = 0.61 (0.11 to 1.10); * p* = 0.027 τ^2^ = 0.028; I^2^ = 18.1%

Publication-bias tests are reported for the overall meta-analysis. In Panel B, dose is defined as occlusion-minutes, calculated as total training minutes multiplied by the opaque duty-cycle fraction

*Abbreviations CI* Confidence interval, *df* degrees of freedom, *SE* Standard error, *k* number of comparison-level effect sizes, *RT* Reaction time, *τ*^*2*^ between-study variance, *I*^*2*^ heterogeneity

Because the prespecified minimum number of dose-informative comparisons for spline modelling was not met (k < 8), restricted cubic spline models were not fitted to avoid unstable non-linear estimates. In a sensitivity analysis including one additional very low-exposure comparison (occlusion-minutes = 4.2; k = 6), the slope remained statistically non-significant (*p* = 0.126), while heterogeneity increased markedly (τ^2^ = 0.717). This indicates that any apparent exposure–response trend is not robust under current reporting limitations. As summarised in Table 5 (Panel C), restricting the dose dataset from k = 6 to the primary subset (k = 5) substantially attenuated heterogeneity (τ^2^ from 1.184 to 0.028; I^2^ from 89.0% to 18.1%). Overall, the current evidence is insufficient to define an optimal SVT dose based on occlusion-minutes alone.

### Publication bias and risk-of-bias profile

For the overall meta-analysis (k = 22), visual inspection of the funnel plot did not suggest clear asymmetry. Egger’s regression intercept test was not statistically significant (intercept = − 0.50; SE = 0.46; t = − 1.09; df = 20; *p* = 0.289; 95% CI − 1.47 to 0.46). Trim-and-fill imputed no missing studies (k_imputed = 0), leaving the pooled estimate unchanged (adjusted g = 0.79; 95% CI 0.39–1.19). Influence diagnostics identified two influential comparisons (Gao_2024 and SmithMitroff_2012). Rechecking data and variances revealed no anomalies. Excluding either reduced the pooled estimate to ~ g = 0.64 and τ^2^ to ~ 0.29–0.30, while preserving a positive effect. Key outputs from publication bias and exposure–response modelling are summarised in Table 5.

Risk of bias assessments are summarised in the traffic-light plots (Figures S4–S5). Among the 14 randomised trials, 6 (43%) were rated low risk and 8 (57%) raised some concerns; none were rated high risk. The three non-randomised studies were judged to be at moderate risk across ROBINS-I domains.

## Discussion

This systematic review and meta-analysis synthesised evidence from 17 controlled studies examining SVT and sport-related outcomes. The overall pooled estimate from a conventional random-effects model indicated a moderate-to-large average benefit (Hedges’ g = 0.79, 95% CI 0.39–1.19). However, heterogeneity was substantial (I^2^ = 84.3%), with a wide prediction interval (− 1.02 to 2.60), indicating considerable variation across sports, outcome tasks, and implementation contexts. A multilevel random-effects model yielded a similar pooled estimate (g = 0.84, 95% CI 0.33–1.34) and indicated that modelled heterogeneity was concentrated predominantly at the between-study level (ICC ≈ 1.00). Taken together, these results suggest that differences across sport context, task design, and programme implementation are likely more influential than the clustering introduced by multiple outcomes within the same study. Accordingly, the pooled estimate should be interpreted as an average effect across heterogeneous designs, rather than a uniform expectation of performance improvement.

A key interpretive issue is exposure timescale. Acute single-session exposures showed no clear effect, whereas multi-session programmes showed a clear benefit (g = 0.88). This distinctionstraints. Fon matters because pooling both yields an overall estimate of stroboscopic exposure, not a pure estimate of retained training adaptation. Accordingly, practical recommendations should be grounded primarily in the multi-session evidence base, while acute findings are better viewed as mechanistic demonstrations of performance under stroboscopic for instance, stroboscopic eyewear applied only during warm-up did not enhance sport-specific reaction speed in highly trained table tennis athlete [64]. This issue also intersects with transfer distance. Many included outcomes were proximal or intermediate tasks that preserved some perceptual or timing demands of sport but reduced movement coupling, task uncertainty, or competitive context. Consistently, the largest pooled effects were observed for perceptual-cognitive outcomes and visuomotor reaction time, with smaller effects for motor speed/reactive agility. This pattern is consistent with the proposed mechanisms of SVT, whereby intermittent visual occlusion perturbs visual input and increases demands on anticipation, cue use, and decision-making, which should map more strongly onto perceptual-cognitive and timing outcomes than onto outcomes dominated by pure movement speed [6, 9, 10, 14, 65, 66]. Nonetheless, substantial heterogeneity – especially within reaction-time outcomes – suggests that task representativeness, training content, and device settings influence observed effects. Transfer frameworks therefore remain central when interpreting the present pooled estimates, as training and testing tasks differ in representativeness and information-movement coupling [22]. Overall, evidence is stronger for near-to-intermediate transfer than for far transfer to representative sport performance.

Our conclusions are constrained by the current evidence base. Several outcome domains are informed by only one to three comparisons, and key stroboscopic parameters were often incompletely described. These features limit moderator interpretation and preclude equally strong claims across all outcome domains or transfer distances. Accordingly, conclusions remain deliberately cautious: the evidence supports potential benefits, but not a universal or context-independent effect.

Exploratory exposure–response modelling based on occlusion-minutes did not provide statistical evidence for an association with effect size in the primary dose-informative subset (5 studies; k = 5; *p* = 0.503). This should not be interpreted as evidence that dose is unimportant, but rather because of limited data and the fact that occlusion-minutes is only a partial proxy for exposure. Occlusion-minutes captures cumulative opaque time but not the temporal structure of the strobe sequence, drill difficulty, stimulus dynamics, information-movement coupling, or decision complexity. Consequently, interventions with similar occlusion-minutes may impose very different perceptual-cognitive demands. In addition, when one very low-exposure comparison was added (k = 6), the slope remained non-significant (*p* = 0.126) and between-study heterogeneity increased sharply, showing that any apparent trends are unstable under current reporting limitations. Future SVT trials should therefore report, at minimum, the device model, exact on/off timing or frequency, duty cycle, progression rules, per-session duration, weekly frequency, total training minutes, derived occlusion-minutes, drill content, stimulus speed, decision demands, testing conditions (with or without stroboscopic occlusion), assessment timing (immediate and delayed), adherence, and adverse effects. Without this level of detail, cumulative exposure–response synthesis will remain both underpowered and conceptually incomplete. This conclusion aligns with previous reviews highlighting incomplete and inconsistent reporting of device parameters and intervention content in the sports-vision literature [16–18, 23, 24].

Publication-bias analyses showed no evidence of small-study effects (Egger *p* = 0.289), and trim-and-fill imputed no missing studies. However, these findings do not eliminate concern about bias because funnel-plot methods can be underpowered when the number of comparisons is modest and heterogeneity is high [45, 46]. In addition, influence diagnostics identified two influential comparisons (Gao_2024 and SmithMitroff_2012). Rechecking their extracted inputs and sampling variances revealed no imputation anomalies, but excluding either reduced the pooled estimate to ~ g = 0.64 and narrowed the prediction interval. This supports a cautious interpretation: the overall direction of effect remains positive, but the magnitude of the pooled estimate is partly shaped by a small number of large effects. Given the difficulty of blinding in stroboscopic interventions, well-matched active controls (for example, sham eyewear or time- and attention-matched drills without occlusion) are especially important for reducing expectancy effects.

Several limitations should be noted. First, some domains included few studies (k = 1–3), which limits the precision of domain-level conclusions. Second, intervention reporting was inconsistent – particularly duty-cycle structure and timing details – constraining exposure modelling and moderator interpretation. Third, heterogeneity was substantial, indicating that effects may depend on sport context, drill design, participant level, task representativeness, control-condition design, and stroboscopic parameters. Fourth, five full-text records could not be obtained at the eligibility stage, so some potentially relevant evidence may have been missed despite the comprehensive search strategy. Finally, most studies were modest in size; therefore, adequately powered trials with preregistered, theoretically grounded outcomes that are explicitly matched to transfer claims remain a priority [17–21]. In addition, some SVT evidence is currently available only in dissertations or non-peer-reviewed reports [67, 68], which increases uncertainty and complicates cumulative synthesis.

Future research should: (1) prespecify transfer distance of each outcome using representativeness-based frameworks [22]; (2) conduct adequately powered randomised controlled trials with well-matched active controls; (3) incorporate delayed follow-up assessments and sport-representative outcomes to distinguish immediate exposure effects from retained training adaptations; (4) experimentally manipulate stroboscopic timing structure, duty cycle, progression, and scheduling variables; and (5) standardise reporting of device parameters and per-session exposure to enable more informative cumulative synthesis [15, 16, 23, 24].

## Conclusions

This meta-analysis supports a moderate-to-large average stroboscopic exposure effect across heterogeneous designs, driven by multi-session programmes and stronger for near-to-intermediate transfer outcomes than for acute exposures or far transfer outcomes. Exploratory dose modelling using occlusion-minutes did not identify a reliable exposure–response association and cannot currently justify an optimal dose recommendation. SVT may be a useful adjunct in sports that rely heavily on rapid perceptual decisions, but stronger reporting standards, well-matched controls, and conceptually grounded transfer assessments are required to establish when, how, and for whom it is effective.

## Supplementary Information

Supplementary Material 1.

## Data Availability

The datasets supporting the conclusions of this article are available in the Zenodo repository (https://doi.org/10.5281/zenodo.17055273).

## Abbreviations

CI: Confidence interval
CR2: Cluster-robust variance estimator (small-sample correction)
df: degrees of freedom
ICC: Intraclass correlation coefficient
I^2^: Inconsistency index
IG/CG: Intervention group/control group
IQR: Interquartile range
MOT: Multiple-object tracking
PI: Prediction interval
PRISMA: Preferred Reporting Items for Systematic Reviews and Meta-Analyses
PROSPERO: International Prospective Register of Systematic Reviews
RA: Reactive agility
RCT: Randomised controlled trial
REML: Restricted maximum likelihood
ROBINS-I: Risk Of Bias In Non-randomised Studies–of Interventions
RoB 2: Cochrane Risk of Bias 2 tool (randomised trials)
RT: Reaction time
SD: Standard deviation
SE: Standard error
SVT: Stroboscopic visual training
τ^2^: Between-study variance
