# Supplementary material for: Effects of stroboscopic visual training on sport performance: a systematic review and meta-analysis with exploratory exposure–response modelling
Source: BMC Sports Sci Med Rehabil. 2026 Jul 21;18:499. doi: 10.1186/s13102-026-01765-y (PMC13673800; doi:10.1186/s13102-026-01765-y)
Supplement: Supplementary file 1 [file 13102_2026_1765_MOESM1_ESM.zip › PRISMA_Checklist_2020_v1.pdf]

| Section/topic | Item # | Checklist item (PRISMA 2020, paraphrased) |
|---|---|---|
| Title | 1 | Identify the report as a systematic review and/or meta-analysis. |
| Abstract | 2 | Provide a structured summary of background, objectives, methods (including eligibility, information sources, and synthesis methods), results, limitations, and conclusions. |
| Introduction | 3 | Describe the rationale for the review in the context of what is already known. |
| Introduction | 4 | Provide an explicit statement of the objective(s) or question(s) being addressed. |
| Methods | 5 | Specify the inclusion and exclusion criteria (PICOS), and how studies were grouped for the syntheses. |
| Methods | 6 | Specify all information sources (databases, registers, websites) and the date last searched. |
| Methods | 7 | Present full search strategies for all databases, including any filters or limits used. |
| Methods | 8 | Specify the methods used to select studies, including how many reviewers screened records and full texts. |
| Methods | 9 | Specify the methods used to collect data from reports, including how many reviewers collected data and how discrepancies were resolved. |
| Methods | 10a | List and define all outcomes for which data were sought. |
| Methods | 10b | List and define all other variables for which data were sought and any assumptions made about missing/unclear information. |
| Methods | 11 | Specify the methods used to assess risk of bias in included studies. |
| Methods | 12 | Specify for each outcome the effect measures used in the synthesis or presentation of results. |
| Methods | 13a | Describe the processes used to decide which studies were eligible for each synthesis. |
| Methods | 13b | Describe any methods required to prepare data for synthesis, such as handling of missing statistics or conversions. |
| Methods | 13c | Describe any methods used to tabulate or visualise results of individual studies and syntheses. |

| | | |
|---|---|---|
| Methods | 13d | Describe any methods used to synthesise results and provide a rationale for the choice(s). |
| Methods | 13e | Describe any methods used to explore possible causes of heterogeneity among study results. |
| Methods | 13f | Describe any sensitivity analyses conducted to assess robustness of the synthesized results. |
| Methods | 14 | Describe any methods used to assess risk of bias due to missing results (reporting biases). |
| Methods | 15 | Describe any methods used to assess certainty or confidence in the body of evidence. |
| Results | 16a | Describe the results of the search and selection process, ideally using a flow diagram. |
| Results | 16b | Cite studies that appear to meet inclusion criteria but were excluded and explain why. |
| Results | 17 | Present characteristics for which data were extracted from each study. |
| Results | 18 | Present assessments of risk of bias for each included study. |
| Results | 19 | For all outcomes, present summary statistics and effect estimates for each study. |
| Results | 20a | For each synthesis, briefly summarise the characteristics and risk of bias among contributing studies. |
| Results | 20b | Present results of all statistical syntheses conducted. |
| Results | 20c | Present results of investigations of heterogeneity among study results. |
| Results | 20d | Present results of sensitivity analyses. |
| Results | 21 | Present assessments of risk of bias due to missing results (reporting biases). |
| Results | 22 | Present assessments of certainty in the body of evidence. |
| Discussion | 23a | Provide a general interpretation of the results in the context of other evidence. |
| Discussion | 23b | Discuss any limitations of the evidence included in the review. |
| Discussion | 23c | Discuss any limitations of the review processes used. |
| Discussion | 23d | Discuss implications for practice, policy, and/or future research. |

| | | |
|---|---|---|
| Other information | 24a | Provide registration information for the review and whether a protocol exists. |
| Other information | 24b | Indicate where the review protocol can be accessed, or state that a protocol was not prepared. |
| Other information | 24c | Describe and explain any amendments to information provided at registration or in the protocol. |
| Other information | 25 | Describe sources of financial or non‑financial support for the review. |
| Other information | 26 | Declare any competing interests of review authors. |
| Other information | 27 | Report the availability of data, code and other materials. |

| Location in manuscript |
|---|
| Title page; Manuscript title |
| Abstract |
| §1 Introduction, ¶1–5 |
| §1 Introduction, final paragraph (Objectives 1–5) |
| §2.3 Eligibility criteria; Table 1 (PICOS) |
| §2.2 Searches; Supplementary File: Search strategies |
| Supplementary File: Search strategies |
| §2.4 Study selection; PRISMA flow (Figure 1) |
| §2.5 Data extraction and coding |
| §2.5 Data extraction (Outcome values) |
| §2.5 Data extraction (coding of moderators, assumptions) |
| §2.6 Risk-of-bias; Figures S4–S5 (robvis) |
| §2.7 Statistical analysis (Hedges' g) |
| §2.3 Eligibility criteria; §2.7.1 Primary pooling |
| §2.5 Data extraction (imputation via Wan et al.) |
| §2.7; Figures 2–4; Table 4–5 |

§2.7.1 Random‑effects (REML) with 95% prediction intervals

§2.7.3 Meta‑regression (RVE, CR2, Knapp–Hartung); §2.7.4 Sensitivity

§2.7.4 Sensitivity (leave‑one‑out; influence; ρ variation)

§2.7.5 Publication bias (Egger; trim‑and‑fill)

§2.8 GRADE (Table S3)

§3.1 Study selection; Figure 1 (PRISMA)

Supplementary File: Full‑text exclusions with reasons

§3.2 Study characteristics; Table 2

§3.7 and Figures 5–6 (robvis); Table S5 sensitivity

Figure 2 (study‑level forest); Table 4

§3.2–3.4; §3.7

§3.3–3.5; Figures 2–4; Table 4

§3.8 Meta‑regression (RVE); §3.6 Influence/sensitivity

§3.6 Sensitivity; Tables S5–S6

§3.7 Publication bias; Figure S7

§2.8; Table S3 (GRADE)

§4 Discussion, ¶1–3

§4 Discussion, Limitations

§4 Discussion, Limitations / Methods caveats

§4 Discussion, Practical implications & Future research

§2.1 Protocol and registration; PROSPERO CRD420251090541

§2.1; Supplementary Appendix A

§2.1 Deviations from protocol (dose–response; acute trials)

Declarations: Funding (none)

Declarations: Competing interests (none)

§2.9 Data availability; Declarations
