# Supplementary material for: Effects of stroboscopic visual training on sport performance: a systematic review and meta-analysis with exploratory exposure–response modelling
Source: BMC Sports Sci Med Rehabil. 2026 Jul 21;18:499. doi: 10.1186/s13102-026-01765-y (PMC13673800; doi:10.1186/s13102-026-01765-y)
Supplement: Supplementary file 1 [file 13102_2026_1765_MOESM1_ESM.zip › Supp01_SearchStrategies_v1.pdf]

Protocol: Registered in PROSPERO (CRD420251090541).

Primary search: 18 July 2025. Update: 19 August 2025.

Limits: none (no language/date/study-type limits).

Concept sets:

- Set A (SVT / intermittent vision / occlusion): stroboscop*, “strobe light”, “strobe glass*”, “strobe eyewear”, “stroboscopic vision”, “intermittent vision”, “visual occlusion”, “occlusion goggle*”, “flicker glass*”, “reduced vision”, “limited vision”, “dynamic visual acuity”, “visual training”, “perceptual training”, device names (Senaptec, Nike Vapor, PLATO).
- Set B (sport / performance outcomes): sport*, athlete*, “athletic performance”, “motor skill*”, “motor performance”, “physical performance”, “reaction time”, agility, coordination, “hand-eye”/“eye-hand” coordination, strength, endurance, balance, sprint*, “movement accuracy”, “skill acquisition”, “visual cognition”.

Note on reporting: Database yields contributing to PRISMA (19 Aug 2025) were: PubMed 340; Web of Science 988; Scopus 673; SPORTDiscus 16; Embase 470; CENTRAL 153.

1) PubMed (MEDLINE via PubMed)

Searched: 18 Jul 2025 (update 19 Aug 2025)

Fields: Title/Abstract + MeSH where applicable

Query (copy-ready):

(

stroboscop*[Title/Abstract] OR "strobe light"[Title/Abstract] OR "strobe glass*"[Title/Abstract] OR

"strobe eyewear"[Title/Abstract] OR "stroboscopic vision"[Title/Abstract] OR

"intermittent vision"[Title/Abstract] OR "visual occlusion"[Title/Abstract] OR

"occlusion goggle*"[Title/Abstract] OR "flicker glass*"[Title/Abstract] OR

"reduced vision"[Title/Abstract] OR "limited vision"[Title/Abstract] OR

"dynamic visual acuity"[Title/Abstract] OR "visual training"[Title/Abstract] OR

"perceptual training"[Title/Abstract] OR Senaptec[Title/Abstract] OR "Nike Vapor"[Title/Abstract] OR PLATO[Title/Abstract]

OR "Vision, Ocular"[MeSH] OR "Perceptual Learning"[MeSH]

)

AND

(

sport*[Title/Abstract] OR athlete*[Title/Abstract] OR "athletic performance"[Title/Abstract] OR

"motor skill*"[Title/Abstract] OR "motor performance"[Title/Abstract] OR "physical performance"[Title/Abstract] OR

"reaction time"[Title/Abstract] OR agility[Title/Abstract] OR coordination[Title/Abstract] OR

"hand-eye"[Title/Abstract] OR "eye-hand"[Title/Abstract] OR strength[Title/Abstract] OR endurance[Title/Abstract] OR

balance[Title/Abstract] OR sprint*[Title/Abstract] OR "movement accuracy"[Title/Abstract] OR

"skill acquisition"[Title/Abstract] OR "visual cognition"[Title/Abstract]

OR "Athletic Performance"[MeSH] OR "Motor Skills"[MeSH] OR "Psychomotor Performance"[MeSH] OR "Reaction Time"[MeSH] OR "Sports"[MeSH]

)

Filters applied: none.

2) Web of Science Core Collection (SCI-EXPANDED, SSCI, A&HCI, ESCI)

Searched: 18 Jul 2025 (update 19 Aug 2025)

Fields: Topic (TS=Title/Abstract/Keywords)

Query:

TS=(

stroboscop* OR "strobe light" OR "strobe glass*" OR "strobe eyewear" OR "stroboscopic vision" OR

"intermittent vision" OR "visual occlusion" OR "occlusion goggle*" OR "flicker glass*" OR

"reduced vision" OR "limited vision" OR "dynamic visual acuity" OR "visual training" OR "perceptual training" OR

Senaptec OR "Nike Vapor" OR PLATO

)

AND

TS=(

sport* OR athlete* OR "athletic performance" OR "motor skill*" OR "motor performance" OR "physical performance" OR

"reaction time" OR agility OR coordination OR ("hand NEAR/1 eye") OR ("eye NEAR/1 hand") OR

strength OR endurance OR balance OR sprint* OR "movement accuracy" OR "skill acquisition" OR "visual cognition"

)

Limits: none.

3) Scopus (Elsevier)

Searched: 18 Jul 2025 (update 19 Aug 2025)

Fields: TITLE-ABS-KEY

Query:

TITLE-ABS-KEY(

stroboscop* OR "strobe light" OR "strobe glass*" OR "strobe eyewear" OR "stroboscopic vision" OR

"intermittent vision" OR "visual occlusion" OR "occlusion goggle*" OR "flicker glass*" OR

"reduced vision" OR "limited vision" OR "dynamic visual acuity" OR "visual training" OR "perceptual training" OR

Senaptec OR "Nike Vapor" OR PLATO

)

AND

TITLE-ABS-KEY(

sport* OR athlete* OR "athletic performance" OR "motor skill*" OR "motor performance" OR "physical performance" OR

"reaction time" OR agility OR coordination OR "hand W/1 eye" OR "eye W/1 hand" OR

strength OR endurance OR balance OR sprint* OR "movement accuracy" OR "skill acquisition" OR "visual cognition"

)

Limits: none.

4) SPORTDiscus (EBSCOhost)

Searched: 18 Jul 2025 (update 19 Aug 2025)

Fields: TI, AB, KW + Subject Headings where available

Query (EBSCO syntax):

(

TI(stroboscop* OR "strobe light" OR "strobe glass*" OR "strobe eyewear" OR "stroboscopic vision" OR

"intermittent vision" OR "visual occlusion" OR "occlusion goggle*" OR "flicker glass*" OR

"dynamic visual acuity" OR Senaptec OR "Nike Vapor" OR PLATO)

OR

AB(stroboscop* OR "strobe light" OR "strobe glass*" OR "strobe eyewear" OR "stroboscopic vision" OR

"intermittent vision" OR "visual occlusion" OR "occlusion goggle*" OR "flicker glass*" OR

"dynamic visual acuity" OR Senaptec OR "Nike Vapor" OR PLATO)

)

AND

(

TI(sport* OR athlete* OR "athletic performance" OR "motor skill*" OR "reaction time" OR agility OR coordination OR

"hand-eye" OR "eye-hand" OR strength OR endurance OR balance OR sprint* OR "movement accuracy" OR

"skill acquisition" OR "visual cognition")

OR

AB(sport* OR athlete* OR "athletic performance" OR "motor skill*" OR "reaction time" OR agility OR coordination OR

"hand-eye" OR "eye-hand" OR strength OR endurance OR balance OR sprint* OR "movement accuracy" OR

"skill acquisition" OR "visual cognition")

)

Limits: none.

5) Embase (Ovid)

Searched: 18 Jul 2025 (update 19 Aug 2025)

Fields/Headings: Emtree exploded where appropriate + multi-purpose fields (mp.)

Line-by-line (Ovid) strategy:

1. (stroboscop* or "strobe light" or "strobe glass*" or "strobe eyewear" or "stroboscopic vision" or

"intermittent vision" or "visual occlusion" or "occlusion goggle*" or "flicker glass*" or

"dynamic visual acuity" or Senaptec or "Nike Vapor" or PLATO).mp.

2. exp sport/ or exp athlete/ or exp athletic performance/ or exp motor performance/ or exp psychomotor performance/

or exp reaction time/ or exp agility/ or exp coordination/ or exp hand eye coordination/ or exp endurance/ or

exp strength/ or exp balance/

3. (sport* or athlete* or "athletic performance" or "motor skill*" or "motor

performance" or "physical performance" or

"reaction time" or agility or coordination or "hand-eye" or "eye-hand" or strength or endurance or balance or

sprint* or "movement accuracy" or "skill acquisition" or "visual cognition").mp.

4. 2 or 3

5. 1 and 4

6. limit 5 to human (optional; not applied in the primary search)

Limits: none (human filter not applied in the primary search to maximise recall).

6) Cochrane CENTRAL (Cochrane Library)

Searched: 18 Jul 2025 (update 19 Aug 2025)

Fields: Title, Abstract, Keyword

Query:

(

stroboscop* OR "strobe light" OR "strobe glass*" OR "strobe eyewear" OR "stroboscopic vision" OR

"intermittent vision" OR "visual occlusion" OR "occlusion goggle*" OR "flicker glass*" OR

"dynamic visual acuity" OR Senaptec OR "Nike Vapor" OR PLATO

)

AND

(

sport* OR athlete* OR "athletic performance" OR "motor skill*" OR "motor performance" OR "physical performance" OR

"reaction time" OR agility OR coordination OR "hand-eye" OR "eye-hand" OR strength OR endurance OR balance OR

sprint* OR "movement accuracy" OR "skill acquisition" OR "visual cognition"

)

Limits: none.

Other sources

- Google Scholar: first 200 titles screened using (“stroboscopic vision” OR “strobe glasses” OR “visual occlusion”) AND (sport* OR athlete*).
- Backward/forward citation chasing via Web of Science.
- Trial registries (ClinicalTrials.gov; ChiCTR) queried in parallel; not included in PRISMA database counts.
