# Supplementary material for: Effects of stroboscopic visual training on sport performance: a systematic review and meta-analysis with exploratory exposure–response modelling
Source: BMC Sports Sci Med Rehabil. 2026 Jul 21;18:499. doi: 10.1186/s13102-026-01765-y (PMC13673800; doi:10.1186/s13102-026-01765-y)
Supplement: Supplementary file 1 [file 13102_2026_1765_MOESM1_ESM.zip › Supp03_GRADE_SummaryOfFindings_v1.pdf]

1. Table S3. GRADE Summary of Findings

Domain	k	N IG	N CG	Pooled g (95% CI)	Certainty
Overall sport-related performance	22	375	376	0.76 (0.39 – 1.13)	Moderate
Perceptual-cognitive tasks	3	37	37	1.23 (0.71 – 1.75)	Moderate
Visuo-motor reaction-time	11	144	143	1.04 (0.21 – 1.88)	Moderate
Motor speed / reactive agility	7	174	176	0.35 (0.06 – 0.64)	Low
Visual tracking / MOT	1	20	20	0.98 (—)	Very low

注：正数 Hedges g 代表频闪组优于对照。CI = 置信区间。

1.1. 降级依据：

a 发表偏倚：Egger t = 2.39, p = 0.03。
b 精确性不足：总样本数 < 300（Overall 阈值 < 400）。
c 单一研究。
d 非随机对照 → 风险偏倚。
e k = 1，无法评估不一致性。
f ≥ 50 % 研究 RoB 为 'some concerns'。
g 95 % CI 跨越最小重要差异 (g = 0.20)。
