# Supplementary material for: Effects of stroboscopic visual training on sport performance: a systematic review and meta-analysis with exploratory exposure–response modelling
Source: BMC Sports Sci Med Rehabil. 2026 Jul 21;18:499. doi: 10.1186/s13102-026-01765-y (PMC13673800; doi:10.1186/s13102-026-01765-y)
Supplement: Supplementary file 1 [file 13102_2026_1765_MOESM1_ESM.zip › Supplementary_File_2_FullText_Exclusions.docx]

# Additional file 3. Supplementary File 2: Full‑text exclusions (summary of reasons)

This file summarises the operational reasons for excluding records at the full‑text eligibility stage. Counts for each reason are reported in the PRISMA flow diagram. The categories below were applied consistently using the prespecified PICOS criteria.

| Exclusion reason category | Operational definition used in this review | Typical examples |
| --- | --- | --- |
| Wrong intervention | The intervention did not involve wearable stroboscopic/shutter eyewear providing intermittent occlusion during task execution, or the stroboscopic component could not be isolated from a multi‑component intervention. | General vision training without intermittent occlusion; VR/AR vision training; multimodal programmes where SVT exposure could not be separated. |
| Wrong comparator | No eligible non‑stroboscopic control condition was available for the primary contrast. | No‑control pre–post studies; comparisons between two different stroboscopic settings without a non‑stroboscopic control. |
| Wrong population | Participants did not meet the prespecified population criteria (healthy athletes/physically active participants in sport‑related contexts). | Clinical/rehabilitation populations; para‑athletes; populations outside the sport/physically active context. |
| Wrong outcome | Outcomes were not plausibly related to sport‑relevant performance, or did not align with the review’s outcome domains. | Purely physiological endpoints without a performance measure; outcomes unrelated to visual/perceptual‑motor performance. |
| Wrong study design | The study was not a controlled trial as prespecified (RCT/quasi‑RCT/controlled trial), or design features prevented extraction of an eligible effect. | Reviews, editorials, qualitative studies; conference abstracts without full methods/data; uncontrolled designs. |
| Insufficient data for effect size computation | The paper did not provide enough quantitative information to compute Hedges’ g (and the information could not be obtained or reconstructed). | Percent change only without dispersion; missing sample sizes; outcomes reported only narratively. |
| Duplicate or overlapping sample | The full text represented duplicate reporting of an included dataset or an overlapping sample where the most complete/appropriate report was retained. | Conference abstract and subsequent full paper; duplicate publications of the same trial. |
| Full text not obtainable | The full text could not be retrieved after reasonable efforts. | Unobtainable records despite library access and author contact attempts. |

Note: If a record met more than one exclusion reason, the primary reason most directly tied to PICOS eligibility was recorded to maintain mutually exclusive PRISMA accounting.
