# Supplementary material for: Effects of stroboscopic visual training on sport performance: a systematic review and meta-analysis with exploratory exposure–response modelling
Source: BMC Sports Sci Med Rehabil. 2026 Jul 21;18:499. doi: 10.1186/s13102-026-01765-y (PMC13673800; doi:10.1186/s13102-026-01765-y)
Supplement: Supplementary file 1 [file 13102_2026_1765_MOESM1_ESM.zip › Supplementary_Table_S2_MissingDataRequests.docx]

# Supplementary Table S2: Missing data requests and author-contact procedures

Purpose: to document how missing quantitative information was handled to enable effect-size computation (Hedges’ g) and to maintain reproducibility. For privacy reasons, the correspondence log (email addresses, message bodies) is archived by the review team and is available from the corresponding author upon reasonable request.

## S2.1 What was considered “missing data”

- Missing data were defined as any of the following information being unavailable from the full text (including tables and appendices):
- sample size per group for the eligible comparison;
- post-intervention (or immediately post-test) mean and a dispersion measure (SD, SE, CI, IQR) for at least one group;
- sufficient statistics to compute a standardised mean difference (e.g., change scores with SD of change, or test statistics with enough information for conversion);
- clear reporting of the eligible time point (≤1 week after intervention endpoint for multi-session studies) and, for cross-over trials, first-period data.

## S2.2 Author-contact procedure

When required information was missing and could not be reconstructed from figures or ancillary files, we contacted corresponding authors by email. Two contact attempts were made (initial request plus one reminder), spaced no more than 7 days apart. Requests were limited to the minimum required information (typically group means/SDs and group sample sizes for the eligible time point).

## S2.3 Hierarchy of resolution rules used when data were incomplete

| Scenario | Resolution rule | Where documented in the manuscript |
| --- | --- | --- |
| Outcome values reported only in a figure (no numerical table) | Digitised the plotted values using WebPlotDigitizer (v4.5) with independent cross-checking; retained the post-test time point closest to intervention end. | Methods (Data extraction); note on digitisation. |
| Dispersion not reported as SD (e.g., SE, CI, IQR) | Converted to SD where possible; when only median/IQR were available, imputed mean/SD using Wan et al. (2014). | Methods (Statistical analysis; imputation rule). |
| Multiple eligible outcomes / multiple SVT arms sharing one control group | Extracted all eligible outcomes by domain; split control-group n evenly across comparisons to avoid double-counting. | Methods (Data extraction and coding). |
| Cross-over trial | Extracted only first-period data to minimise carryover bias. | Methods (Eligibility; data handling). |
| Dose parameters incomplete for occlusion-minutes computation | Retained comparison for overall meta-analysis but excluded from occlusion-minutes exposure–response modelling. | Methods (Exposure metric); Results (dose subset definition). |

## S2.4 Note on transparency

The above rules were applied consistently across studies. Any remaining non-extractable outcomes were excluded from quantitative synthesis and are reflected in the PRISMA accounting. The review team retains the original extraction worksheets and correspondence records for audit purposes.
