# Supplementary material for: Effects of stroboscopic visual training on sport performance: a systematic review and meta-analysis with exploratory exposure–response modelling
Source: BMC Sports Sci Med Rehabil. 2026 Jul 21;18:499. doi: 10.1186/s13102-026-01765-y (PMC13673800; doi:10.1186/s13102-026-01765-y)
Supplement: Supplementary file 1 [file 13102_2026_1765_MOESM1_ESM.zip › Additional_file_1_Supplementary_Appendix_A_Protocol_Deviations.docx]

# Additional file 1. Supplementary Appendix A: Protocol and post hoc deviations

This appendix summarises the a priori protocol, key analytic decisions, and any deviations made after protocol registration. The purpose is transparency and reproducibility (PRISMA 2020).

## A1. Registration and protocol status

The review protocol was developed a priori and prospectively registered on PROSPERO (CRD420251090541) on 5 January 2025. The protocol specified the research question, eligibility criteria (PICOS), screening procedures, primary analysis approach, risk-of-bias tools, and sensitivity analyses. The final manuscript reports any deviations below.

## A2. Research question and objectives (as prespecified)

The overarching question was: in athletes or physically active participants, does stroboscopic visual training (SVT) improve sport-related performance outcomes compared with a non-stroboscopic control?

Prespecified objectives were to:

- estimate the overall effect of SVT on sport-related outcomes using random-effects meta-analysis;
- estimate domain-specific effects for: (i) perceptual–cognitive tasks, (ii) visuomotor reaction time, (iii) motor speed / reactive agility, and (iv) visual tracking / multiple object tracking (MOT);
- clarify the estimand by separating acute single-session exposures from multi-session programmes;
- evaluate methodological quality using RoB 2 for randomised trials and ROBINS-I for non-randomised comparisons;
- assess the certainty of evidence with GRADE.

## A3. Eligibility criteria (PICOS; as prespecified)

Participants: healthy adolescents or adults (≥10 years) who were athletes or physically active participants in sport-related contexts.

Interventions: wearable stroboscopic/shutter eyewear providing intermittent occlusion during task execution. Both multi-session programmes (≥1 week) and single-session acute exposures were eligible for overall synthesis, with explicit stratification by exposure timescale.

Comparators: non-stroboscopic control conditions (e.g., sham/transparent eyewear, normal vision, or conventional training without occlusion).

Outcomes: perceptual–cognitive, visuomotor, and sport-related performance outcomes with sufficient data to compute Hedges’ g.

Study design: RCTs, quasi-RCTs, and controlled trials. For cross-over designs, only first-period data were used.

## A4. Prespecified data handling rules

- Effect sizes were coded as Hedges’ g, with positive values indicating better performance with SVT. For outcomes where lower values indicate better performance (e.g., reaction time), effects were sign-reversed.
- When a study contributed multiple eligible comparisons sharing a single control group, the control-group sample size was split evenly across comparisons to avoid double-counting.
- For studies reporting medians/ranges or medians/IQRs, means and standard deviations were imputed using Wan et al. (2014) when needed.
- For cross-over trials, only first-period data were extracted to minimise carryover bias.

## A5. Prespecified primary analyses

Primary synthesis used a conventional random-effects meta-analysis (REML) with heterogeneity reported as τ² and I². A 95% prediction interval was planned for the overall conventional random-effects model.

Because some trials contributed multiple comparisons across domains, a multilevel random-effects model was planned as a robustness check to account for within-study dependence.

## A6. Post hoc deviations and rationale

Two protocol deviations were made and are explicitly labelled as exploratory or estimand-clarifying analyses:

1. Deviation 1: Exploratory exposure–response modelling was added.

Rationale: device parameters and cumulative exposure are inconsistently reported in the SVT literature. To explore whether reported exposure could explain heterogeneity, we derived an exposure proxy (occlusion‑minutes = total training minutes × opaque duty‑cycle fraction) when the required information was extractable. Because occlusion‑minutes does not capture timing structure (e.g., frequency progression) or task constraints, this analysis was interpreted strictly as exploratory and hypothesis-generating.

2. Deviation 2: Acute single-session trials were included in overall pooling but interpreted as mechanistic evidence.

Rationale: acute exposures can test short-term performance effects under intermittent vision but do not necessarily reflect retained training adaptation. Therefore, acute trials were retained in the overall estimate (to describe the overall stroboscopic exposure effect) and were analysed separately (acute vs multi-session) to clarify the estimand. Acute trials were excluded a priori from exposure–response modelling.

## A7. Exposure–response analysis decision rules (exploratory)

Dose modelling was restricted to multi-session programmes and to the reaction-time subset with extractable occlusion‑minutes. Non-linear associations were planned using restricted cubic splines (three knots at the 10th, 50th, and 90th percentiles). Spline models were fitted only when the number of dose-informative comparisons was sufficient (prespecified k ≥ 8) and knots were distinct; otherwise, a linear meta-regression was reported to avoid unstable non-linear inference.

## A8. Reporting notes

All deviations are described in the Methods section of the manuscript and are reflected in the supplementary materials. The review team maintained the direction-harmonisation rule (positive g indicates SVT superiority) across all outcomes.
