# Supplementary material for: Effects of stroboscopic visual training on sport performance: a systematic review and meta-analysis with exploratory exposure–response modelling
Source: BMC Sports Sci Med Rehabil. 2026 Jul 21;18:499. doi: 10.1186/s13102-026-01765-y (PMC13673800; doi:10.1186/s13102-026-01765-y)
Supplement: Supplementary file 1 [file 13102_2026_1765_MOESM1_ESM.zip › Supp06_FINAL.zip!/Supp06/Multi/baujat_Multi.pdf]

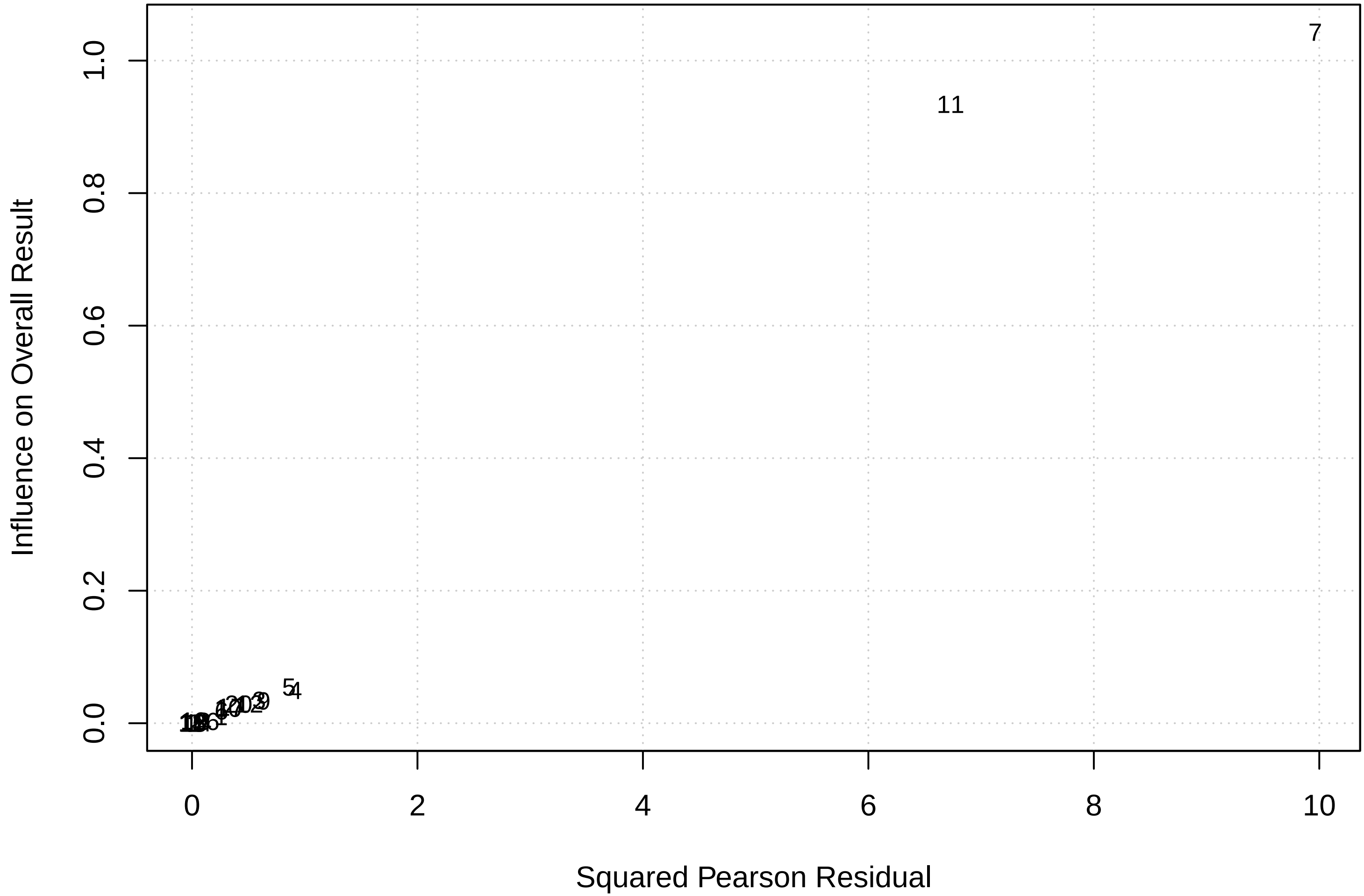

Influence on Overall Result
Squared Pearson Residual
0.0
0.2
0.4
0.6
0.8
1.0
0
2
4
6
8
10
7
11
5
4
