# Supplementary material for: Effects of stroboscopic visual training on sport performance: a systematic review and meta-analysis with exploratory exposure–response modelling
Source: BMC Sports Sci Med Rehabil. 2026 Jul 21;18:499. doi: 10.1186/s13102-026-01765-y (PMC13673800; doi:10.1186/s13102-026-01765-y)
Supplement: Supplementary file 1 [file 13102_2026_1765_MOESM1_ESM.zip › Supp06_FINAL.zip!/Supp06/Multi/forest_Multi.pdf]

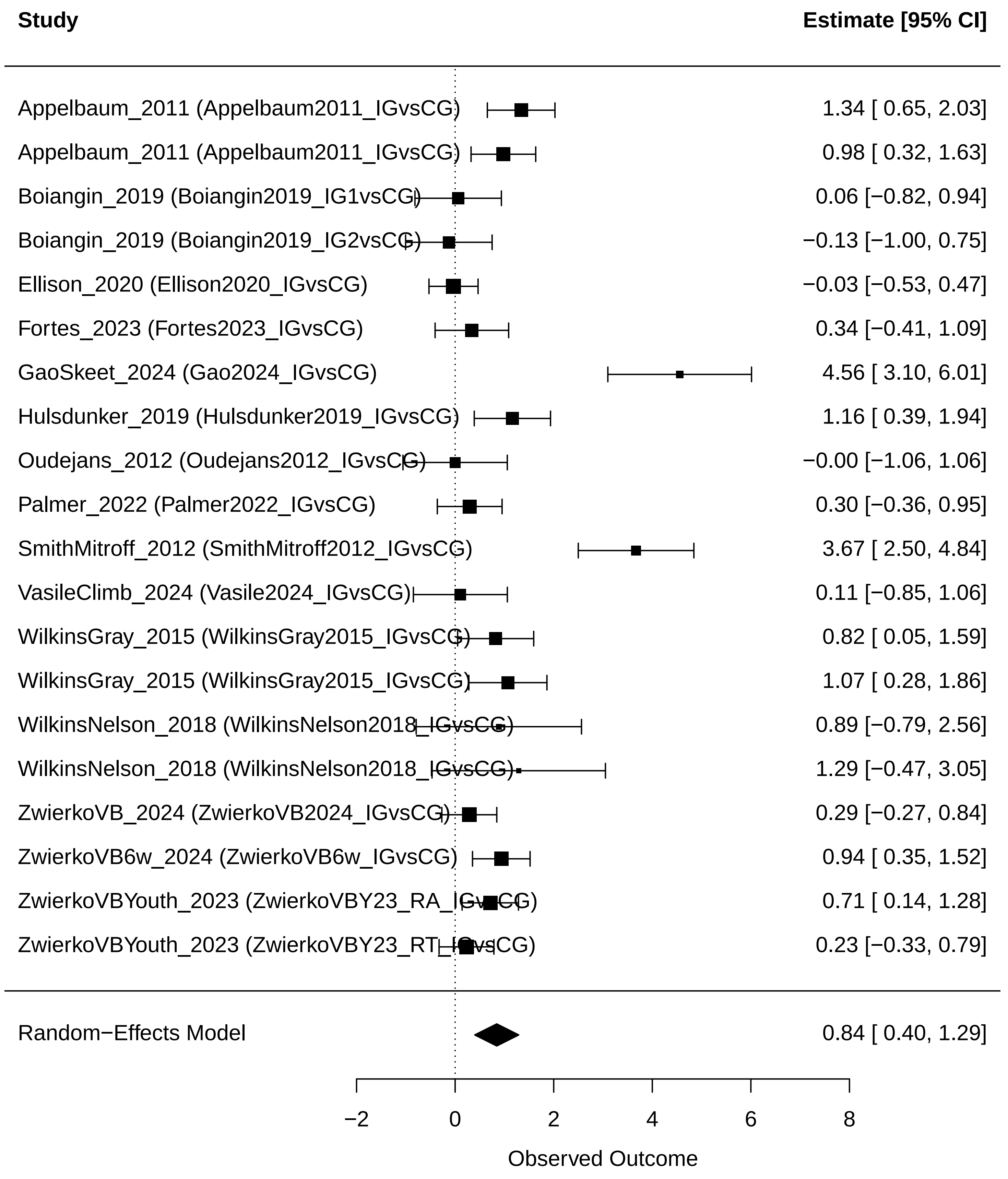

Study
Estimate [95% CI]
Appelbaum_2011 (Appelbaum2011_IGvsCG)
1.34 [ 0.65, 2.03]
Appelbaum_2011 (Appelbaum2011_IGvsCG)
0.98 [ 0.32, 1.63]
Boiangin_2019 (Boiangin2019_IG1vsCG)
0.06 [−0.82, 0.94]
Boiangin_2019 (Boiangin2019_IG2vsCG)
−0.13 [−1.00, 0.75]
Ellison_2020 (Ellison2020_IGvsCG)
−0.03 [−0.53, 0.47]
Fortes_2023 (Fortes2023_IGvsCG)
0.34 [−0.41, 1.09]
GaoSkeet_2024 (Gao2024_IGvsCG)
4.56 [ 3.10, 6.01]
Hulsdunker_2019 (Hulsdunker2019_IGvsCG)
1.16 [ 0.39, 1.94]
Oudejans_2012 (Oudejans2012_IGvsCG)
−0.00 [−1.06, 1.06]
Palmer_2022 (Palmer2022_IGvsCG)
0.30 [−0.36, 0.95]
SmithMitroff_2012 (SmithMitroff2012_IGvsCG)
3.67 [ 2.50, 4.84]
VasileClimb_2024 (Vasile2024_IGvsCG)
0.11 [−0.85, 1.06]
WilkinsGray_2015 (WilkinsGray2015_IGvsCG)
0.82 [ 0.05, 1.59]
WilkinsGray_2015 (WilkinsGray2015_IGvsCG)
1.07 [ 0.28, 1.86]
WilkinsNelson_2018 (WilkinsNelson2018_IGvsCG)
0.89 [−0.79, 2.56]
WilkinsNelson_2018 (WilkinsNelson2018_IGvsCG)
1.29 [−0.47, 3.05]
ZwierkoVB_2024 (ZwierkoVB2024_IGvsCG)
0.29 [−0.27, 0.84]
ZwierkoVB6w_2024 (ZwierkoVB6w_IGvsCG)
0.94 [ 0.35, 1.52]
ZwierkoVBYouth_2023 (ZwierkoVBY23_RA_IGvsCG)
0.71 [ 0.14, 1.28]
ZwierkoVBYouth_2023 (ZwierkoVBY23_RT_IGvsCG)
0.23 [−0.33, 0.79]
Random−Effects Model
0.84 [ 0.40, 1.29]
−2
0
2
4
6
8
Observed Outcome
