# Supplementary material for: Effects of stroboscopic visual training on sport performance: a systematic review and meta-analysis with exploratory exposure–response modelling
Source: BMC Sports Sci Med Rehabil. 2026 Jul 21;18:499. doi: 10.1186/s13102-026-01765-y (PMC13673800; doi:10.1186/s13102-026-01765-y)
Supplement: Supplementary file 1 [file 13102_2026_1765_MOESM1_ESM.zip › Supp06_FINAL.zip!/Supp06/Acute/funnel_Acute.pdf]

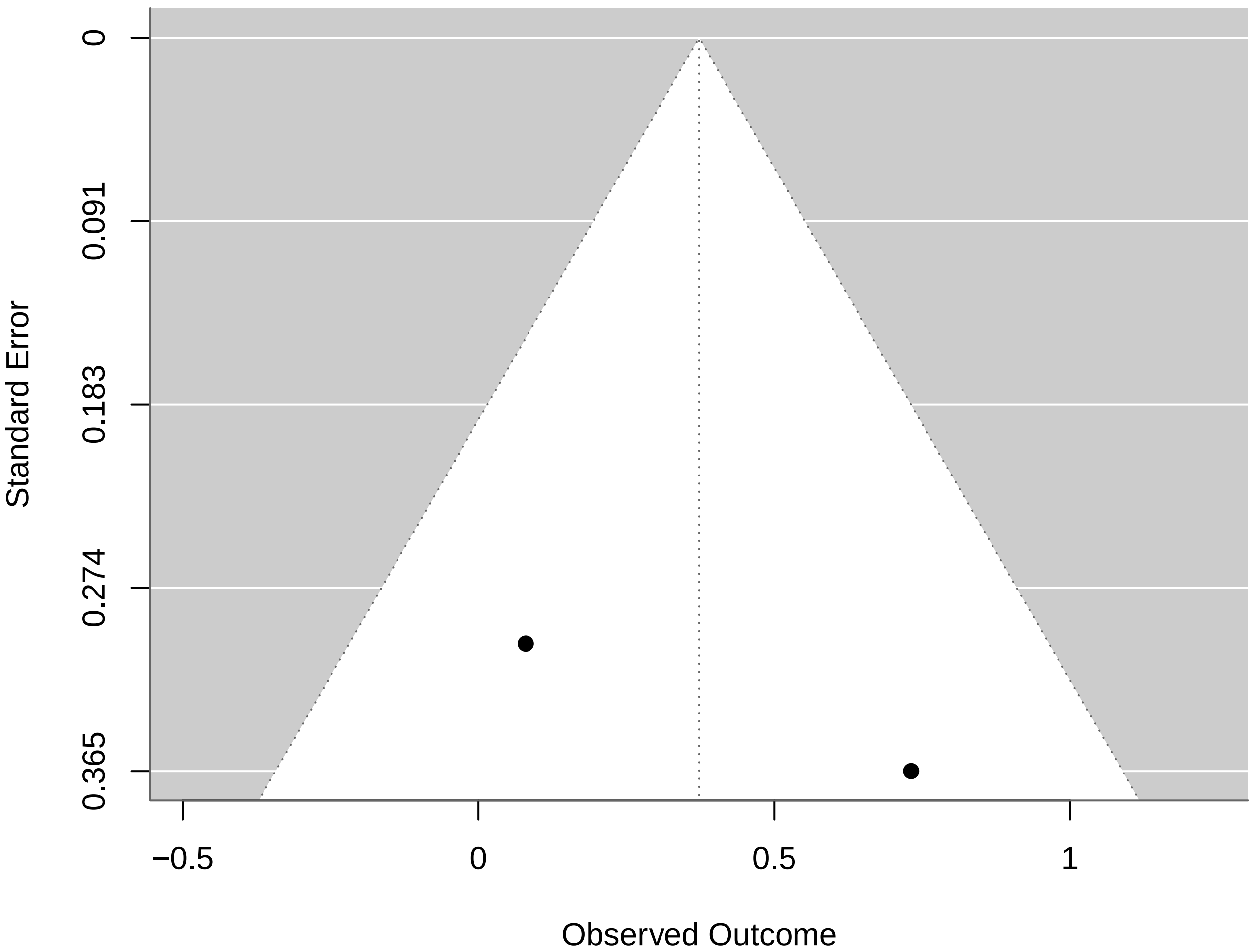
Standard Error
0
0.091
0.183
0.274
0.365
−0.5
0
0.5
1
Observed Outcome
