# Supplementary material for: Effects of stroboscopic visual training on sport performance: a systematic review and meta-analysis with exploratory exposure–response modelling
Source: BMC Sports Sci Med Rehabil. 2026 Jul 21;18:499. doi: 10.1186/s13102-026-01765-y (PMC13673800; doi:10.1186/s13102-026-01765-y)
Supplement: Supplementary file 1 [file 13102_2026_1765_MOESM1_ESM.zip › Supp06_FINAL.zip!/Supp06/Acute/forest_Acute.pdf]

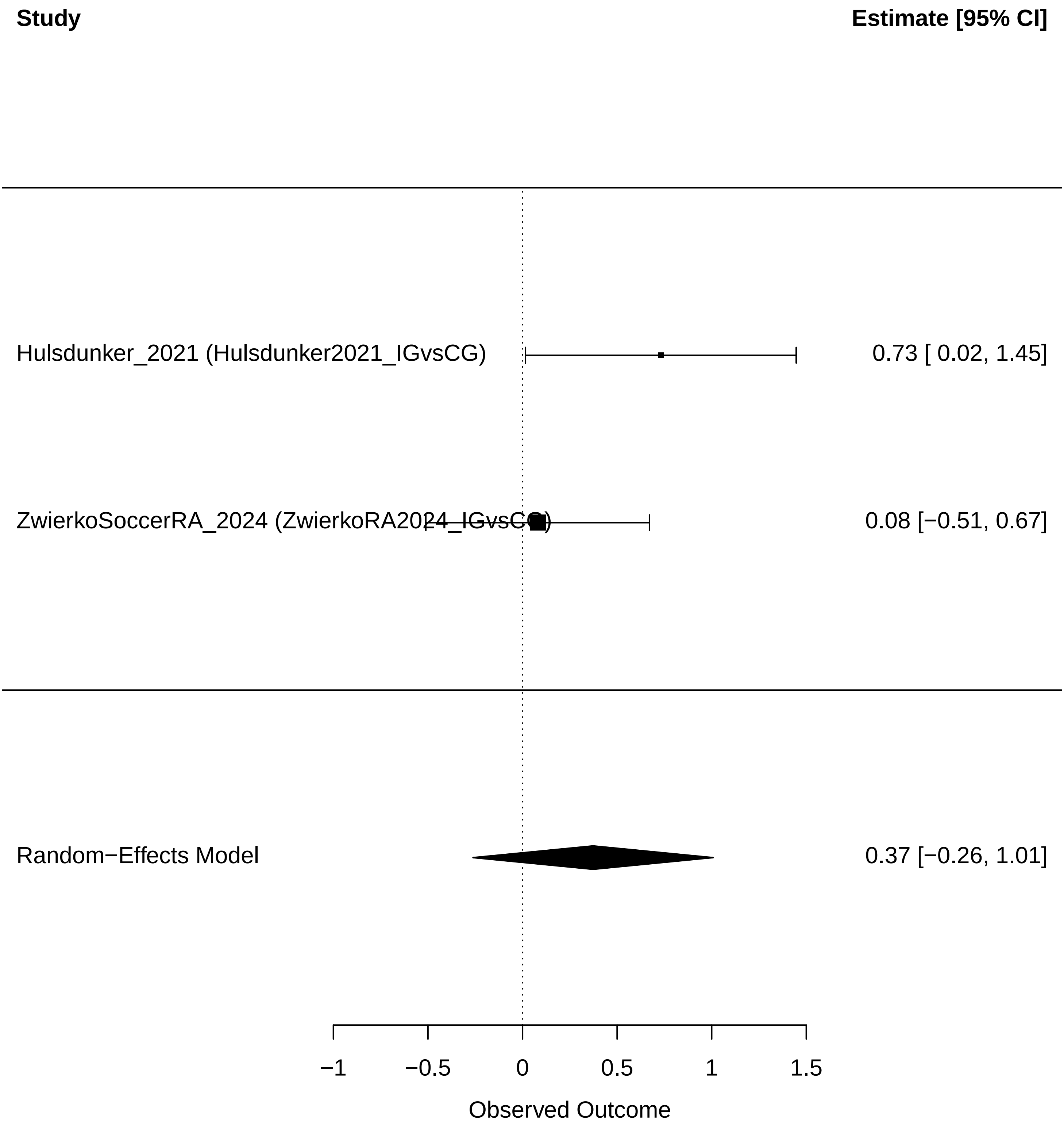
Study
Estimate [95% CI]
Hulsdunker_2021 (Hulsdunker2021_IGvsCG)
0.73 [0.02, 1.45]
ZwierkoSoccerRA_2024 (ZwierkoRA2024_IGvsCG)
0.08 [−0.51, 0.67]
Random−Effects Model
0.37 [−0.26, 1.01]
−1
−0.5
0
0.5
1
1.5
Observed Outcome
